# A Comprehensive Overview of Targeted Therapy in Metastatic Renal Cell Carcinoma

**DOI:** 10.2174/156800912802429265

**Published:** 2012-09

**Authors:** Z Mihály, Z Sztupinszki, P Surowiak, B Győrffy

**Affiliations:** 1 Research Laboratory for Pediatrics and Nephrology, Hungarian Academy of Sciences - Semmelweis University 1st Dept. of Pediatrics; 2 Department of Histology and Embryology, Wrocław University School of Medicine, ul. Chałubińskiego 6a, 50-356 Wrocław, Poland

**Keywords:** Biomarkers, everolimus, renal cell cancer, sunitinib, temsirolimus, tyrosine kinase inhibitors.

## Abstract

Chemotherapy and immunotherapy failed to deliver decisive results in the systemic treatment of metastatic renal cell carcinoma. Agents representing the current standards operate on members of the RAS signal transduction pathway. Sunitinib (targeting vascular endothelial growth factor), temsirolimus (an inhibitor of the mammalian target of rapamycin - mTOR) and pazopanib (a multi-targeted receptor tyrosine kinase inhibitor) are used in the first line of recurrent disease. A combination of bevacizumab (inhibition of angiogenesis) plus interferon α is also first-line therapy. Second line options include everolimus (another mTOR inhibitor) as well as tyrosine kinase inhibitors for patients who previously received cytokine. We review the results of clinical investigations focusing on survival benefit for these agents. Additionally, trials focusing on new agents, including the kinase inhibitors axitinib, tivozanib, dovitinib and cediranib and monoclonal antibodies including velociximab are also discussed. In addition to published outcomes we also include follow-up and interim results of ongoing clinical trials. In summary, we give a comprehensive overview of current advances in the systemic treatment of metastatic renal cell carcinoma.

## INTRODUCTION

Renal cell carcinoma (RCC) is responsible for about 2-3% of all malignant diseases in adults. Clinical signs do not develop early due to the location of the tumor, consequently the overall five-year survival is as low as 20-25%. The most important feature in the selection of the appropriate therapy is the presence of metastases. The primary treatment is surgery ranging from partial nephrectomy of localized RCCs to cytoreductive nephrectomy in extended tumors with multiple metastases. Then, for advanced, metastatic or recurrent disease a systemic therapy can be administered. As RCCs are generally resistant to chemo- and radiotherapy, this systemic management consist of the administration of targeted therapy agents. 

In this context, the recent advance of agents targeting signal transduction pathways was inevitable. While normal cells maintain a considerable diversity in the intracellular signaling pathways, tumor cells may develop an intrinsic dependence on regulation defects in some of the most important pathways enabling constitutive growth – this phenomenon was termed oncogene addiction [1]. In cancer cells, one of the most frequently activated pathways is the signaling upstream and downstream of Ras. Ras signaling impinges on cytoplasmic and nuclear targets *via *its numerous downstream effectors and pathways. These signaling pathways regulate the organization of the actin cytoskeleton, cell survival, cell cycle progression and gene expression (for a recent review of Ras regulated genes see [2]). It has already been reported that these agents are significantly involved in the pathogenesis of metastatic RCC (mRCC) [3], and currently influencing the Ras signaling represents the most up-to-date treatment options for RCC.

## AGENTS RECOMMENDED IN CURRENT TREATMENT REGIMENS

We surveyed the literature of the RCC treatment using PubMed (http://www.pubmed.com), the abstracts of the last five ASCO Meetings (http://chicago2012.asco.org/Past AnnualMeetings.aspx), the online databases of NCCN (http://www.nccn.org) and those of Clinical Trials (http://clinicaltrials.gov). When searching, the key words of "kidney", "renal", "cancer", "clinical trial", "survival" as well as the names of the agents were used.

In Fig. (**1**) we have summarized the Ras signal transduction pathway including targets of agents of actual treatment protocols for RCC. First line systemic treatment includes the oral, small-molecule, multi-targeted receptor tyrosine kinase inhibitors (TKI) sunitinib, sorafenib and pazopanib, the intravenous mTOR inhibitor temsirolimus and the oral mTOR inhibitor everolimus. Furthermore, a monoclonal antibody – Bevacizumab - combined with IFN – is also approved as first line therapy in RCC.

### Sunitinib – the New Gold Standard

Sunitinib was approved by the FDA (Food and Drug Administration) for RCC therapy as a continuous once-daily dosing regimen in 2006. It is recommended as first-line treatment in advanced and stage IV RCC by the 2012 NCCN (National Comprehensive Cancer Network) and by the 2010 EAU (European Association of Urology) guidelines. 

The results of the first decisive study in which the effectiveness of sunitinib therapy was observed were published in 2006 by Motzer and his colleagues. Here, in 106 cytokine resistant metastatic RCC (mRCC) patients the progression-free survival (PFS) was 8.3 (7.8 to 14.5) months [4], and led to the accelerated FDA approval of sunitinib for mRCC treatment. Following up this study, the latest results in clinicaltrials.gov show 9.5 months (33.9 to 58.4 weeks) PFS and 26 months (61.0 to 133.1 weeks) overall survival (OS) benefits besides 33% (24.2 to 42.8) objective response rate (ORR) (www.clinicaltrials.gov, trial no. NCT00077974).

According to a 2009 phase III trial comprising 750 treatment naïve mRCC patients randomly receiving sunitinib or IFN as first-line therapy, sunitinib treated patients’ overall survival is significantly longer (26.4 months (95% confidence interval (CI): 23.0 to 32.9 months) than IFN treated patients’ (21.4 months (CI: 17.9 to 26.9 months)) [5]. For that study, the PFS results were already published in 2007 showing 11 (95% CI: 9-13) versus 5 (CI: 4-6) months in favor of sunitinib. Interestingly, a small difference was measured in assessing the PFS by core radiology (48.3 weeks, CI: 46.4 -58.3) and by the investigators assessment (47.7 weeks, CI: 46.3-58.1) [6]. Currently, the ongoing study shows 28.7 months (CI: 100.1-142.9 weeks) OS besides 38.7% ORR in case of sunitinib treated mRCC patients (trial no.: NCT00083889).

An even higher response rate was documented in the ongoing phase IV SUNIKA study (trial no.: NCT00460798) with 40.6% ORR in first-line sunitinib treated patients with advanced or metastatic RCC. The ORRs were also similar and quite high in the NCT00254540 study where patients in the first-line achieved 48%, and pretreated patients 46.2% ORRs. In this phase II study (trial no. NCT00254540), the OS results in first-line sunitinib-patients compared to sunitinib after cytokine treatment were quite similar: 35.8 months (13.7 to 149.3 weeks) and 35.25 months (17.0 to 141.1 weeks). The PFS results show some difference, as in the first line sunitinib-treated cohort it was 13.25 months (3.9 to 130.0 weeks), while in the pretreated group it was only 11.5 months (10.0 to 124.1 weeks). The result of another study in phase II (trial no. NCT00338884) shows an ORR of 35% in first-line sunitinib treated patients with advanced RCC and a PFS of 9 (5.6 to 11.1) months. 

Researchers reported in an abstract in the ASCO2010 Annual Meeting for patients receiving sunitinib longer than 2 years (28 patients from 109) an OS of 45 (39 to 51) months besides manageable toxicity^1^. As a consequence of surgical intervention, one must consider the long-term side effects of sunitinib treatment in patients on hemodialysis. The estimated median PFS in this cohort was 15 months and the median OS 29 (12 to 47) months. These results show that sunitinib treatment in patients on hemodialysis is feasible and well tolerated. 

The circadian effects of the administration were compared in a phase II study (trial no. NCT00137423) in mRCC patients. Here, sunitinib seemed to be nearly equally efficient in patients who got it AM or PM as second line therapy. The PFS of 35.7 (27.6 to 50.0) and 35.3 weeks (20.3 to 38.1) and OS of 91.4 (69.4 to 115.4) and 76.4 (59.6 to 108.3) weeks were similar. However, quite surprisingly, the 28.3% ORR (16.8 to 42.3) was twice as high in case of AM dose as for the PM dose (11.5%, 4.4 to 23.4). According to the published results of this study, the sunitinib treated patients’ PFS was 8.2 (6.4 to 8.4) months and their OS was 19.8 (16.2 to 24.9) months after 26.4 months median follow-up besides an ORR of 20% (12.8% to 28.9%)^2^. 

Due to its manageable safety profile sunitinib is also approved in cytokine-resistant patients as second-line mRCC therapy. Meanwhile, sunitinib in second-line after bevacizumab-based treatment is not advised as category 1 therapy. The results of a phase II trial show 23% (13.2 to 35.5) ORR, 7.6 months (18.3 to 36.7 weeks) PFS and 11.8 months (36.9 to 69.7 weeks) OS (trial no.: NCT00089648).

In a new phase I/II trial (trial no. NCT00113529) the combination of sunitinib with gefitinib has been examined and similar efficacy was found as compared to sunitinib monotherapy with an acceptable safety profile [7]. According to their results in clinicaltrials.gov the ORR was 37.1% (21.5 to 51.1), and the PFS was measured to reach 12.1 months (28.1 to 84.1 weeks).

All these results led to the rapid spreading of sunitinib use since 2006. However, the initial results of Motzer *et al., *were questioned as the investigator assessment of the response rates was nearly 10% higher than the response rate assessed by an independent third-party core imaging laboratory. Therefore, a centralized and independent review of radiological images was suggested for clinical trials [8]. The optimum sunitinib dose is yet to be determined [9]. Moreover, it was mentioned by Stadler *et al., *that the standard radiologic imaging and Response Evaluation Criteria in Solid Tumors (RECIST), which were used to predict the PFS, was suggested to be arbitrary and have never been considered to be a true surrogate end point in this disease. Therefore the calculation of PFS for sunitinib-treated patients might not be reliable. Furthermore, the improvement in progression-free survival does not prove that there was a true benefit [10]. Another commenter remarked that the statistical significance of the improvement seems to be marginal and that the effect of sunitinib on overall survival might almost be certainly underestimated [11]. All of these discussions call for additional clinical trials to objectively verify the effectiveness of sunitinib in RCC treatment.

### Sorafenib – a Dual-Specificity TKI

Sorafenib is a dual-specificity tyrosine kinase inhibitor (TKI) also inhibiting raf, a member downstream of ras. Sorafenib was the first targeted agent to receive approval by the FDA shortly before sunitinib in December 2005. It is recommended as category 1 by the NCCN for cytokine resistant patients. It is recommended only as category 2A in first line and second line (after TKI treatment) therapy in RCC. 

Escudier and his colleagues reported the results of the TARGET phase III clinical trial (NCT00073307) in 2007. For sorafenib in second-line treatment of patients already given one systemic treatment in the previous 8 months they reported a raised PFS to 5.5 months and the OS to 19.3 months compared to placebo (PFS: 2.8 months; OS: 15.9 months). Disease progression was observed in 9% of sorafenib and in 30% of placebo treated patients. However, the treatment was associated with increased toxic adverse effects [12]. A second analysis of the TARGET results demonstrated advantage in OS after sorafenib treatment (17.8 months) compared to post-cross-over placebo (14.3 months). Additionally, the level of VEGF was proved as a prognostic marker of PFS and OS in these patients [13]. 

In a phase III study (NCT00586105), patients who got sorafenib after no more than one prior systemic therapy, the PFS was 5.4 (4.1 to 7.4) months and the OS was 7.8 (0.9 to 13.4) months. The results of another phase II study in which 95 Japanese patients with advanced or metastatic RCC are followed, show 12.7 month (386 days (274 to 502)) PFS in sorafenib treated patients after 45 months of follow-up (NCT00586495).

According to the latest results of a phase II trial, patients tolerating a sorafenib dose above 400 mg appeared to have greater clinical benefit, as the median PFS in these patients was 3.7 (1.8 to 9.5) months. Patients receiving 600 mg or 800 mg sorafenib had a median PFS of 7.4 (6.3 to 12.0) and 8.5 (5.6 to 14.9) months, while no clinically relevant differences were observed in the severity or frequency of adverse side effects^3^. According to another phase II study the use of higher dose of sorafenib could be a valid option for patients with progression after the failure of VEGF and TKI therapy^4^.

The most up-to-date results for sorafenib were demonstrated in an abstract at the 2011 ASCO Annual Meeting. In this study the effectiveness of the sorafenib plus everolimus combination was assessed in advanced RCC patients. The regimen was active and tolerable, however benefit versus sequential single agents was not observed^5^.

Although the results of the TARGET trial led to the approval of sorafenib by the FDA, Twardowski and his colleagues questioned this decision because the approval is valid for adults with advanced renal cell carcinoma, which is larger population compared to the relatively small proportion of mRCC patients representing the population involved in the study [14]. Moreover, in a phase II trial the use of sorafenib as first-line therapy in RCC resulted in similar PFS as IFN (however, sorafenib-treated patients experienced greater rates of tumor size reduction, better Quality Adjusted Life Years (QUALY), and improved tolerability) [15]. According to several studies, the effectiveness of axitinib^6^ and everolimus in sunitinib-resistant mRCC patients may be superior to sorafenib^7^.

### Pazopanib

The third tyrosine kinase inhibitor pazopanib is administered once daily orally and was cleared by the FDA as first-line therapy in RCC patients in 2009. Pazopanib has a higher selectivity than that of the other tyrosine kinase inhibitors [16] and has a remarkable VEGFR-2 inhibitory potential [17]. In a phase II study pazopanib achieved 35% (28 to 41) ORR in patients with advanced RCC (31% in treatment naïve population and 37% in pretreated population) and it was generally well tolerated in treatment-naïve and IFN or bevacizumab-pretreated patients. The median PFS was 52 weeks (44 to 60 weeks), versus just 45 weeks (36 to 59 weeks) of PFS in 28 patients who were randomly assigned to placebo [18].

In a phase III study patients were divided into four groups including treatment naïve, cytokine-pretreated, placebo, and pazopanib-treated patients. The median PFS was significantly prolonged in the pazopanib cohort (9.2 months) compared to placebo (4.2 months). In the treatment-naïve subpopulation the median PFS was 11.1 months on pazopanib therapy while the PFS in the placebo-administered patients was only 2.8 months. In patients receiving pazopanib as second-line therapy, the median PFS was 7.4 months outperforming 4.2 months in the matched placebo-treated group. The objective response rate to pazopanib treatment was 30% compared to 3% in placebo [19].

However, lower efficacy of pazopanib was observed in patients who have already been treated by another targeted therapy regimen. According to the latest results of a trial where 44 patients have been treated with pazopanib (32 of them had first line sunitinib and 12 of them had previous bevacizumab treatment), the median PFS was 9.3 months after 9 months follow-up, and the total ORR was only 20%^8^.

In an indirect comparison of the diverse agents used in RCC the PFS was higher with pazopanib compared to IFN alone and it was similar to sunitinib and bevacizumab plus IFN while the grade 3 and 4 adverse effects were the lowest in the pazopanib-treated patients^9^. 

As about 10% of the Caucasian population are homozygous for the UGT1A1 allele, the UGT1A1 genotyping was approved in 2005 for irinotecan to avoid dose-dependent life-threatening neutropenia [20]. Interestingly, Xu and his colleagues reported pazopanib-induced hyperbilirubinemia in patients having the UGT1A1 polymorphism [21]. It remains an open question to assess to what extent these detoxifying mechanisms could contribute to drug resistance and whether they have an influence on the overall survival after pazopanib administration.

### Temsirolimus

Temsirolimus is a specific inhibitor of mTOR, a downstream member of the RAS/PI3K/PKB pathway. It is the only drug recommended as category 1 therapy for RCC patients with poor prognosis. NCCN defines poor prognosis in case at least three out of six risk factors of short survival are present. These include a serum lactate dehydrogenase level of more than 1.5 times the upper limit of the normal range, a hemoglobin level below the lower limit of the normal range, a corrected serum calcium level of more than 10 mg per deciliter (2.5 mmol per liter), the surpassing of a year between randomization and initial diagnosis, a Karnofsky performance score of 60 or 70, and the metastases in multiple organs [22]. In 2007, the intravenously administered temsirolimus became the third drug (and the first mTOR inhibitor) to be approved by the FDA for the treatment of advanced RCC. 

The decision was backed by the results of the Global Advanced Renal-Cell Carcinoma trial. In this phase III trial temsirolimus treatment resulted in improved overall survival of 10.9 (8.6 to 12.7) months in patients outperforming 8.9 (6.1 to 8.8) months of IFN monotherapy. The combined administration of temsirolimus plus IFN compared to the patients in the two other arms did not result in improved OS (8.4 months). The mTOR inhibitor resulted in the best median PFS assessed by investigators on site (temsirolimus: 3.8 months; IFN: 1.9 months; Combination: 3.7 months) as well as by independent investigators in a smaller set of patients (temsirolimus: 5.5 months; IFN: 3.1 months; combination: 4.7 months). The ORR was the lowest in case of IFN therapy (4.8%; 1.9 to 7.8), while the patients on temsirolimus therapy showed 8.6% (4.8 to 12.4) ORR [22]. According to an abstract of the 2007 ASCO Annual Meeting an update analysis of the study examined the relationship between tumor histology, prognostic factors and survival in patients who received either temsirolimus or IFN. Median OS and progression-free survival times were longer in patients receiving temsirolimus than patients receiving IFN regardless of tumor histology or patient age. Interestingly, the improvement was the most pronounced in a subset of patients having non-clear cell histology^10^. According to an abstract of the 2010 ASCO Annual Meeting in patients with advanced RCC the median PFS was 141 days (4.7 months) after temsirolimus therapy^11^.

Unfortunately, there are no data about the influence of temsirolimus in not poor prognosis patients, therefore additional studies will be needed for a comprehensive assessment of temsirolimus efficacy in these groups.

### Everolimus

Everolimus, another derivative of sirolimus, is an immunsuppressant mTOR inhibitor approved by the FDA in 2009 for patients with advanced RCC after progression following treatment with sunitinib or sorafenib. It is the only one Category 1 recommendation after TKI therapy in patients with mRCC. An advantage of everolimus over temsirolimus is its oral administration leading to higher compliance. 

According to a study involving 410 RCC patients progressing after VEGF inhibitor therapy, treatment with everolimus prolonged PFS compared to placebo in conjunction with best supportive care. The median PFS was 4.0 (3.7 to 5.5) months in case of everolimus treated patients versus 1.9 (1.8 to 1.9) months after placebo treatment, while the OS was nearly equal in both cohorts (less than 8.8 and 8.8 months) [23]. In a blind follow-up study a persistent and clinically relevant prolongation of PFS to 4.5 months was achieved. An independent central review reported a median PFS of 4.9 (4.4 to 5.5) months^12^. The most recent records of this study show a PFS of 5.42 (4.30 to 6.32) months of the everolimus-treated patients who received only one VEGF-TKI treatment previously. On the other hand, the median PFS was 3.78 (3.25 to 5.13) months in the everolimus group in patients who received two prior VEGFR-TKI treatments^13^. Subsequent treatment after the failure of at least one TKI (sunitinib or sorafenib or bevacizumab) is associated with a promising PFS of 5.1 months after everolimus treatment. We must note, however, that such subsequent therapy was given to patients who achieved superior PFS response to initial TKI exposure. The median OS for patients with or without post-everolimus treatment were 28.2 and 21.2 months respectively^14^.

While the prolonged PFS after everolimus was proved by Motzer *et al., *in 410 patients, its ability to improve OS is still questioned. As the published data showed QUALY equivalent between the everolimus and placebo groups, PFS is also questioned to be a valid surrogate marker of survival in this study [24].

Due to the differences in molecular targets, the combination of mTOR and VEGF inhibitors might improve treatment response in advanced RCC. However, in a phase I trial of simultaneous everolimus and sunitinib administration in RCC patients the combination was associated with significant toxicities and was only tolerated at attenuated doses [25]. Meanwhile, a combination of bevacizumab and everolimus showed in only 19% of the patients grade 3/4 toxicities. This combination was reported to raise median PFS of mRCC patients to 9 months after sunitinib or sorafenib treatment^15^. The results of a study in phase II show limited efficacy and unexpectedly high toxicity of the combination therapy containing bevacizumab and temsirolimus in mRCC [26].

### Axitinib

Axitinib is a small molecule multi-target tyrosine kinase inhibitor approved by the FDA in January 2012 as second line therapy for the treatment of patients with advanced RCC after sunitinib or sorafenib failure. Previously, in the phase III AXIS trial the value and safety of axitinib in second line was confirmed in 723 mRCC patients. An overall ORR of 19% and PFS of 6.7 months were achieved - these were significantly longer compared to sorafenib (PFS: 4,7 months, ORR:11% by investigator assessment) ^16^ [27]. Furthermore, axitinib showed activity in patients with cytokine-refractory metastatic RCC in a phase II trial (PFS: 15.7 (8.4-23.4) months, median OS: 29.9 (20.3-not estimable) months, investigator-assessed ORR: 44.2% (30.5-58.7)) [28]. In another phase II study focusing on sorafenib-refractory mRCC, the ORR was only 22.6%, the median PFS was 7.4 (6.7 to 11.0) months and the median OS was 13.6 (8.4 to 18.8) months [29]. Quite surprisingly, in a cohort of Japanese patients with cytokine-refractory mRCC axitinib therapy delivered an ORR of as high as 55% and a median PFS of 12.9 months^17^.

The AXIS trial was the first to show differences between targeted agents in increasing PFS in pretreated patients. We must note however the non-blinded trial design and the still maturing OS data. Patients without hypertension and with high tolerance in the axitinib group were allowed to increase their doses, whereas those in the sorafenib group were not. As the size of some subgroups was small, no meaningful conclusions can be drawn for the bevacizumab and temsirolimus pretreated cohorts [27]. As the long median PFS noted for axitinib after cytokine failure might eventually correspond to an encouraging outcome in treatment-naïve RCC, Bex and his colleagues proposed the use of axitinib in first-line mRCC treatment [30].

### Bevacizumab Plus IFN

Inhibition of VEGF driven angiogenesis has been demonstrated to prevent vascularisation, thereby suppressing tumor growth. The antiangiogenic antibody, bevacizumab was approved by the FDA for colon cancer in 2004 and subsequently received clearance for other cancer types including RCC in 2009. The current NCCN guideline recommends bevacizumab plus IFN as first–line therapy in RCC as Category 1 and Category 2B after TKI failure.

The approval succeeded the AVOREN trial where the efficacy of bevacizumab plus IFN treatment was assessed in 649 patients. The median PFS was significantly longer in the bevacizumab plus IFN group than it was in the IFN plus placebo group (10.2 months versus 5.4 months). The ORR was higher in case of bevacizumab plus IFN versus only IFN (31% and 13%, respectively), while the OSs were similar (23.3 versus 21.3 months) [31]. 

Similarly, in a phase III trial (CALBG) with 732 patients, OS favored the bevacizumab treatment combined with IFN therapy (8.5 months) compared to IFN monotherapy (5.2 months). ORR was also higher in the bevacizumab plus IFN arm (25.5% vs. 13.1%). However, the toxicity was also higher in the combination-treated group [32]. The latest update of this study mentions 18.3 (16.5 to 22.5) months OS in the bevacizumab cohort compared to 17.4 (14.4 to 20.0) months in the monotherapy arm. While OS favored the bevacizumab plus IFN arm, the difference was not significant. According to their finding, hypertension may be a biomarker of outcome for the bevacizumab plus IFN combination [33].

In the BEVLiN study the safety and efficacy of bevacizumab with low-dose IFN are prospectively assessed in 146 mRCC patients. The trial was designed to allow a cross-trial, descriptive comparison with AVOREN subgroups. The ORR was 22% and the median PFS was 15.6 months in BEVLiN versus 10.5 (10.1 to 12.9) months in the matched AVOREN subgroup. In the low-dose IFN + bevacizumab arm the incidence of IFN-associated adverse effects appears to be reduced compared to the matched AVOREN control group ^18^. A meta-analysis of CALBG and AVOREN results demonstrated that the bevacizumab plus IFN prolonged both OS and PFS as first line therapy for mRCC, especially for MSKCC intermediate risk group ^19^.

The results of the AVOREN and CALGB trials were heavily criticized, as based on the preliminary results the investigators changed the primary end point from OS to PFS. Although the estimated OS for the control group in the AVOREN study was anticipated to be approximately 13 months, an improved patient selection, early diagnosis, supportive care and second-line therapies resulted in an OS of 19.8 months [34]. An abstract of the 2010 ASCO Annual Meeting questioned the use of PFS as a preliminary hint for foretelling OS based on the results of CALBG trial, because the association between PFS and OS was not significant (p=0.52). The median survival time among patients who experienced progression at 3 months was compared to patients who did not progress at 3 months. After the comparison, patients who did not progress at 3 and at 6 months had adjusted hazard ratios of 2.6 for death and those who progressed at 3 and 6-months had 2.9 ^20^.

An important side effect of bevacizumab therapy is the increased grade >3 toxicity (hypertension, anorexia, fatigue, and proteinuria) that was reported in 80% of patients receiving bevacizumab plus IFN compared to 63% of patients receiving IFN monotherapy. One must note however that in patients receiving combination therapy the average duration of treatment was 4 months longer than IFN-α monotherapy patients [33]. Finally, in a large comprehensive meta-analysis, bevacizumab treatment was also associated with a significantly increased risk of arterial thromboembolic events^21^.

Among the highly anticipated combination therapies are the combined use of bevacizumab and a tyrosine kinase inhibitor or an mTOR inhibitor. Due to disappointing phase I results the combination of bevacizumab and sunitinib was discontinued. The particular reasons for this were the high degree of hypertension and vascular and hematologic toxicities at the highest dose level [35]. Similarly, doses were reported not to be long-term tolerable for the combination of bevacizumab and sorafenib in patients with advanced RCC who had not received prior VEGF TKI therapy [36]. In another phase II trial the activity of the bevacizumab – erlotinib combination in mRCC was assessed and found to be well tolerable, although without providing additional clinical benefit as compared to bevacizumab alone^22^.

### Interleukin-2

Although slowly fading out, the current NCCN guidelines still list the high dose IL-2 treatment as a first line treatment option with a category 2A designation. Previously, only IL-2 and IFN could provide increased survival for mRCC patients, as the tumor is generally chemotherapy-resistant. However, according to Negrier and his colleagues IL-2 provides no survival benefit in metastatic renal cancers of intermediate prognosis while inducing a significant risk of toxicity. They also suggest the use of angiogenesis inhibitors, as the achieved PFS was only 3.4 (2.9 to 5.8) and the OS was 15.3 (13.3 to 20.0) months in patients treated by IL-2 [37].

Meanwhile, in a 2010 ASCO Annual Meeting abstract, RR of 29% was achieved in a trial with high dose IL-2, which is significantly higher than historical values. The median PFS was 4.4 months in the 120 patients investigated^23^.

In Table **1** and **2** we summarized the agents and the most important clinical results. Additionally, the structures of the listed tyrosine kinase inhibitors are depicted in Fig. (**2**).

## AGENTS UNDER INVESTIGATION

Besides the above described drugs, a number of trials are currently enroute to confirm or reject potential new agents for systemic therapy of mRCC. None of these agents is currently recommended. The success of sunitinib has lead to the development of several kinase inhibitors, and these represent the most numerous group.

### Kinase Inhibitors

*Tivozanib *is a selective inhibitor of the vascular endothelial growth factor (VEGF) receptors 1, 2 and 3. Tivozanib is intended to substitute current therapies in the first line treatment of patients with advanced RCC. In a phase II trial (NCT00502307) comparing tivozanib and placebo, and median PFS for tivozanib treated patients was 12.5 months for patients with clear cell component and 14.8 for patients who have undergone nephrectomy. An abstract of the TIVO-1 phase II trial (NCT01030783) reported 27.2 % ORR in patient with RCC in 2009^24^. Here, in 272 patients the median PFS and ORR were 11.7 months and 30%. In patients with nephrectomized clear cell RCC, tivozanib demonstrated an increased PFS of 14.8 months and an ORR of 36% with acceptable safety profile^25^. A phase III trial is currently in progress to compare tivozanib with sorafenib in advanced RCC^26^. In TIVO-1, a phase 3 clinical trial evaluating the efficacy and safety of tivozanib compared to sorafenib in 517 patients with advanced RCC Tivozanib demonstrated a statistically significant improvement in PFS with a median PFS of 11.9 months compared to a median PFS of 9.1 months for sorafenib in the overall study population. Furthermore, tivozanib demonstrated significant improvement in PFS (12.7 months) compared to a median PFS of 9.1 months for sorafenib in the pre-specified subpopulation of patients who were treatment-naïve (approximately 70% of the total study population) (http://www.aveopharma.com). The investigators promise presentation of detailed findings from TIVO-1 at the 2012 ASCO. Moreover, tivozanib is the first tyrosine kinase inhibitor to be safely combined with an mTOR inhibitor (temsirolimus) at full dose and schedule of both agents [38]. Interestingly, to this date no study has been initiated to directly compare the efficacy of bevacizumab and tivozanib.

*Dovitinib* demonstrated inhibition of VEGFR and FGFRs in clinical trials. According to the results of a phase II trial the median PFS and OS were 6.1 months and 10.2 months, respectively. Dovitinib treatment was suggested to be a feasible alternative for heavily pre-treated mRCC patients ^27^. An ongoing phase III trial (NCT01223027) is in progress but still without any preliminary results. We must note a publication describing fulminant acneiform eruption after the administration of dovitinib in RCC [39]. 

Other orally administered multi-kinase inhibitors currently in evaluation include *Regorafenib* (BAY 73–4506), a multi-kinase inhibitor tested in a phase II trial administered for previously untreated patients (NCT00664326)^28^, and *Linifanib* which is administered after the failure of a previous TKI therapy. Linifanib is also in a phase II trial (NCT00486538) where the ORR was 9.4% by RECIST, the median PFS was 5.4 months, and the median OS was 13.3 months^29^.

*Cediranib* is a highly potent and selective VEGF signaling inhibitor. Three phase II clinical trials are underway to evaluate the efficacy of Cediranib in metastatic renal cell carcinoma patients (trial no. NCT00303862, NCT00227760, NCT00423332). According to the results of a trial shown at the ASCO 2008 Annual Meeting the median PFS was 8.7 months and 6-month progression-free proportion was 63% in patients with advanced untreated RCC^30^. 

### Monoclonal Antibodies

Monoclonal antibodies are specific antibodies made by identical immune cells that are all clones of a unique parent cell. Currently, bevacizumab is the only FDA approved monoclonal antibody in renal cancer, but a few additional ones are in clinical trials.

*Volociximab* is a chimeric monoclonal antibody against α5β1 integrin inducing apoptosis in the endothelial cells and thereby hampering vascular formation. It was well tolerated in a multicenter phase II study in 40 patients with metastatic clear cell RCC. One patient achieved a partial response while 32 subjects had stable disease for 2 to 22 months. Fourteen (35%) patients had a median PFS of 4 months (range 5.8-22 months) and OS rate at 22 months was 68%^31^. 

*Naptumomab estafenatox* (ABR 217620) is a fusion protein consisting of an antigen-binding fragment from a cancer cell binding antibody that targets metastasis-associated 5T4 and a bacterial superantigen, which is thought to bind to T-cells [40]. Naptumomab estafenatox had specific antitumor activity in cell culture and xenograft models and already passed phase I studies in advanced NSCLC [41]. A phase 2/3 study of naptumomab estafenatox in combination with interferon alpha as a treatment for advanced renal cell carcinoma is in progress (trial no. NCT00420888).

Programmed death-1 (PD-1) is an inhibitory receptor expressed on activated T cells. Previously, the level of immune cells expressing PD-1 was reported to increase in 263 patients with high-risk tumors, and PD-1 has been suggested as a prognostic marker in RCC [42]. One trial with *antiPD-1* (MDX-1106) already reached phase II in patients with poor prognosis and reported high tolerability and evidence of antitumor activity [43].

### Other Agents

*AMG 386* inhibits angiogenesis by sequestering angiopoietin-1 and -2, and preventing their interaction with the Tie2 receptor on endothelial cells. There are two ongoing studies on combination with sunitinib or sorafenib, but so far it did not improve PFS compared to sorafenib plus placebo^32^.

The combination of *gemcitabine* (a nucleoside analogue) and *capecitabine* (a prodrug of 5-fluorouracil) has been studied in several phase II trials in patients with mRCC who received immunotherapy or targeted therapy or underwent prior nephrectomy. Response rates have ranged from 8.4% to 15.8%, median progression-free survival from 4.6 to 7.6 months, and median OS from 10.4 to 23 months. The most common adverse effects were grade 3 or 4 neutropenia in 45-85% of the patients. Interestingly, one of the studies also revealed that patients with the best response were more likely to have a decreased expression of PTEN and an increased expression of mTOR [44]. Meanwhile, in a recent trial with 1006 treatment-naïve patients diagnosed with advanced mRCC, interferon alfa-2a alone or in combination with interleukin-2, and *5-fluorouracil* were assessed, but there was no OS difference between the groups [45]. 

Several studies have investigated the combination oral 5-FU and *thalidomide* in mRCC patients after nephrectomy and progression after IL-2 and interferon treatment. While showing clinical response with tolerable side effects [46-49] novel phase II trials [50] couldn’t confirm the efficacy of thalidomide [50, 51]. A former phase II study recommended further trials for *lenalidomide* (CC-5013), a structural derivative of thalidomide with antiangiogenic and immunomodulatory effects [52]. However, no objective responses were observed later [53]. Ongoing phase I/II trials investigate the efficacy of lenalidomide in combination with sunitinib (NCT00975806), everolimus (NCT01218555), and bevacizumab, sorafenib, temsirolimus, or 5-Fluorouracil, leucovorin, and oxaliplatin (trial no. NCT01183663).

*AS1411* is a DNA aptamer binding nucleolin, its mechanism of action is not yet fully uncovered [54]. According to the result of a phase II trial in the treatment of 33 patients with mRCC, the independently assessed median PFS was 3.9 months with grade 1-3 adverse effects (fatigue and constipation) [55].

*HSPPC-96* (vitespen, Oncophage) was a promising autologous, tumor-derived HSP gp96-peptide complex for cancer immunotherapy. However, it did not delivered any noticeable benefit for patients with metastatic RCC: in a phase II study the median survival was merely 584 days in the HSPPC arm [56]. There were several other vaccine-focused trials initiated for RCC between 1997 and 2007 [57] but their number declined with the emergence of the TKIs. 

Finally, the addition of *13-cis-retinoic acid* (13-CRA) to interferon alfa-2a increased median time to progression from 3.4 to 5.1 months and the median OS from 13.2 to 17.3 months in a phase II/III trial in mRCC patients. Out of 320 patients 162 received IFN therapy alone and 159 of them were given IFN in combination with 13-CRA. The PFS data did not differ dramatically between the two groups (p=0.048). Improvement in efficacy in the combined arm was accompanied by increased, though not serious, toxicity (23% of patients stopped therapy in combinations arm due to toxicity) [58].

## CONCLUSION

The most prominent trend in the RCC clinical trials is the initial introduction of new agents in second line treatment what is then followed by advance into first line. In the near future, we can expect agents currently administered only in second line (Everolimus, Axitinib) to enter first line. Another major tendency is the development of multi-target TKIs: new agents in phase II/III studies, like Regorafenib, Linifanib, Brinavib, Dovitinib, generally have several simultaneous targets. Of these, we can expect agents focusing on genes previously untouched (Dovitinib, Brinavib) to have the fastest approval for a sub-cohort of patients.

However, not only the targets, but also the indications are widely overlapping for current agents. Therefore, the selection of beneficiary population for each available agent must be improved using specific biomarkers. Generally, biomarkers can be used before therapy to estimate response or survival of a specific patient to a specific treatment compared with another treatment [59]. While there is currently no accepted predictive molecular biomarker in kidney cancer treatment, a large set of genes have been already proposed. In Table **3** we have summarized the molecular biomarkers suggested for RCC. The parallel integration of clinical trials and functional genomics datasets might help identify the most promising biomarkers also suitable for clinical use [60].

At the same time we must also note some disadvantages of the recent trials. Most importantly, as there are no clear consensus criteria set for mRCC, the individual trials display high heterogeneity. Not only the trial endpoints, such as overall survival, progression-free survival and overall response rate are different among the studies, but their assessment is not always independent either. Moreover, the pre-treatment conditions - largely because of the recent advance of the targeted agents suppressing previous cytokine protocols - also dramatically differ among the various studies. Finally, in almost all studies the reporting of results is restricted to one or two endpoints. We should require the publication of more results for individual studies in order to enable other investigators to use the results in meta-analyses of subsequent studies. 

We must consider the costs related of the targeted therapy regimens. The incremental cost for a life-year gained is 70,000 pounds for sorafenib [61], 67k USD for sunitinib [62] and 90,000 pounds for temsirolimus [63]. Being relatively expensive alternatives, the disease-related-group (DRG) financing technique most widely used in the US and Europe limits the application of these agents. Therefore, a global breakthrough can only be expected once the generic drugs will enter the market, which is expected in December 2020 for sunitinib and also end of 2020 for sorafenib.

As the overall response rates are still far from ideal, other targeted molecular approaches must be developed in addition to targeting tyrosine kinases. A fresh example for such new mechanisms might be oxidative stress, which is strongly involved in inflammation and carcinogenesis [64]. Renal cell carcinoma patients have increased oxidative stress, which can be effectively alleviated by curative resection [65]. In a recent study, a novel mechanisms linking AMPK and Nox4 to inflammation-induced RCC metastasis was identified. The pharmacological activation of AMPK and/or antioxidants targeting Nox4 have been suggested as a relevant therapeutic intervention to reduce IL-6- and IL-8-induced inflammation and thus invasion in RCC [66].

Targeted therapy agents are not cytotoxic but cytostatic – this leads to the unavoidable development of resistance and to progression. Future research will be needed to identify collateral pathways involved in the development of resistance and to establish newly designed targeted agents leading to higher response rates and longer progression-free survival.

## Figures and Tables

**Fig. (1). An overview of the signal transduction pathways targeted in the systemic treatment of metastatic renal cell carcinoma.**
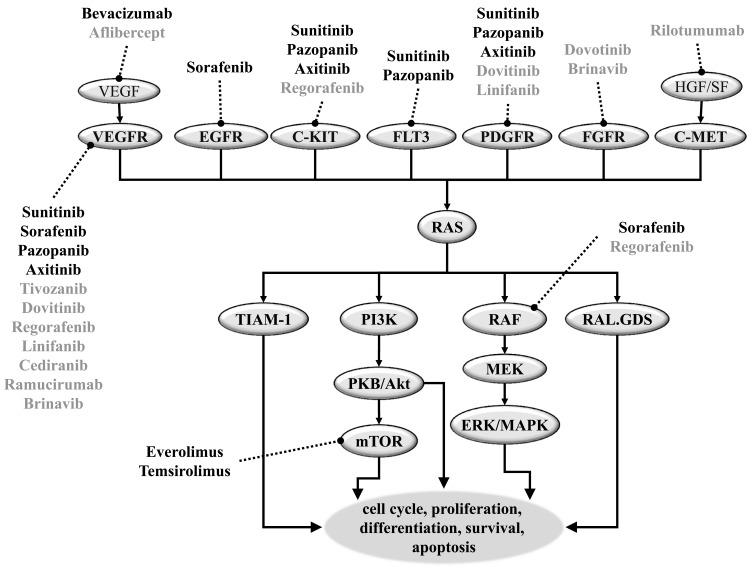
**Abbreviations**: EGF: Endolthelian growth factor, ERK/MAPK: Extracellular signal-regulated kinase/mitogen-activated, FGF: Fibroblast growth factor; FLT3: Fms-like tyrosine kinase receptor-3; GFR: Growth factor receptor, HGF/SF: Growth and motility factor hepatocyte growth factor/scatter factor; mTOR: Mammalian target of rapamycin, MEK: Mitogen-activated protein kinase /extracellular, PDGF: Platelet-derived growth factor; PI3K: Phosphatidylinositol-3-Kinase, RAL.GDS: Ral guanine nucleotide dissociation stimulator, RAS: Rat sarcoma, TIAM-1:T-lymphoma invasion and metastasis 1; VEGF:Vascular endothelial growth factor.

**Fig. (2):**
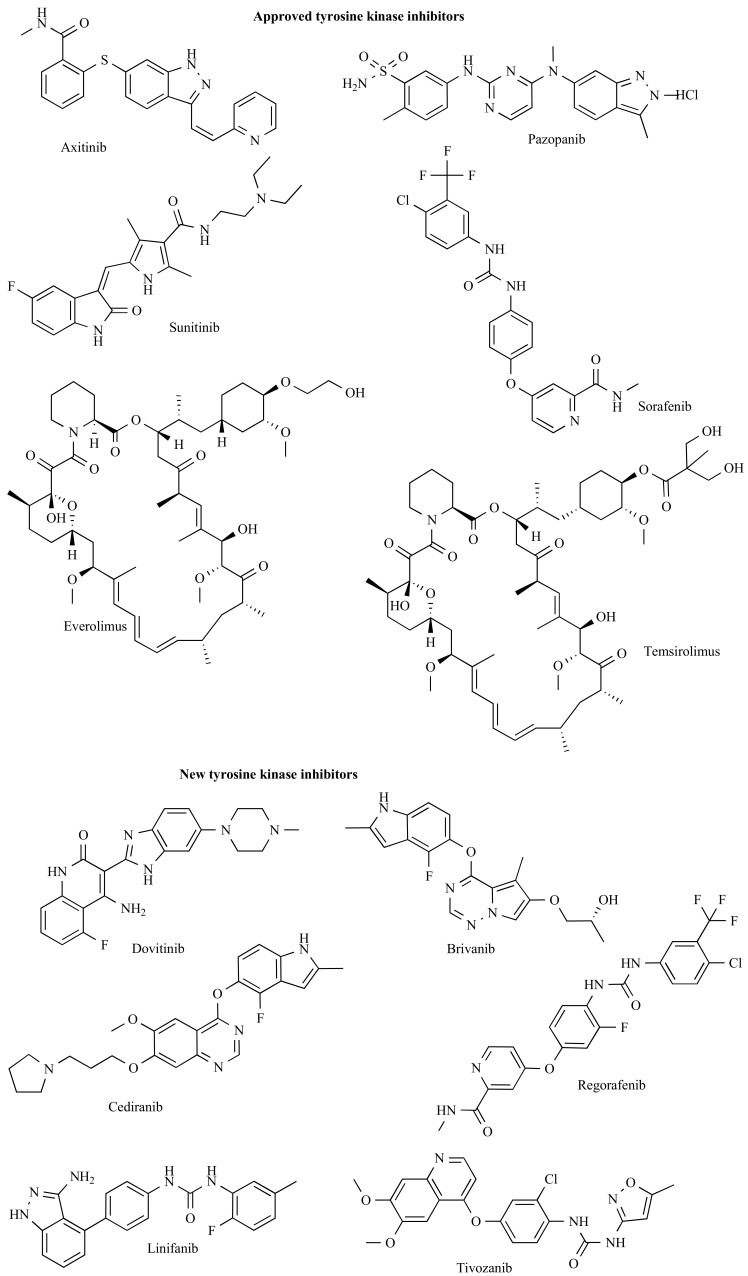
Molecular structures of the tyrosine kinase inhibitors.

**A:** 

Trade name & Supplier	Agent	Approved by FDA		Mechanism	Target	Adverse effects in RCC
*Nexavar® by Bayer and Onyx Pharmaceuticals*	Sorafenib	advRCC	2005	SMI	VEGFR, PDGFR, RAF	fatigue, weight loss, rash, desquamation, hand-foot skin reaction, alopecia, diarrhea, anorexia, nausea and abdominal pain
		HCC	2005			
*Sutent® by Pfizer*	Sunitinib	advRCC,	2006	SMI	FLT3, c-KIT, PDGFR, VEGFR, CSF1R, RET	fatigue, asthenia, fever, diarrhea, nausea, mucositis/stomatitis, vomiting, dyspepsia, abdominal pain, constipation, hypertension, peripheral edema, rash, hand-foot syndrome, skin discoloration, dry skin, hair color changes, altered taste, headache, back pain, arthralgia, extremity pain, cough, dyspnea, anorexia, and bleeding
		GIST	2006			
		PNET	2011			
*Torisel® by Pfizer*	Temsirolimus	advRCC	2007	SMI	mTOR, HIF-1, HIF-2, VEGF	anorexia (incidence ≥30%) and rash, asthenia, mucositis, nausea, edema
*Avastin® by Genetech*	Bevacizumab	mCRC	2004	Mab	VEGF	exfoliative dermatitis (incidence ≥10%) and epistaxis, headache, hypertension, rhinitis, proteinuria, taste alteration, dry skin, rectal hemorrhage, lacrimation disorder, back pain
		GBM	2004			
		NSCLC	2006			
		mRCC	2009			
		mBC	2011			
*Afinitor® by Novartis*	Everolimus	advRCC,	2009	SMI	mTOR, HIF-1, VEGF	diarrhea (incidence ≥30%) and stomatitis, infections, asthenia, fatigue, cough
		PNET	2010			
		SEGA	2011			
*Votrient® by Glaxosmithkline*	Pazopanib	advRCC	2009	SMI	VEGFR, FLT3, c-KIT, PDGFR	diarrhea, hypertension, hair color changes, nausea, anorexia, vomiting
*Inlyta® by Pfizer*	Axitinib	advRCC	2012	SMI	VEGFR, PDGFR, c-KIT	diarrhea, hypertension, fatigue, decreased appetite, nausea, dysphonia, palmar-plantar erythrodysesthesia (hand-foot) syndrome, weight decreased vomiting, fatigue, decreased appetite, nausea, dysphonia, palmar-plantar, asthenia, and constipation

**B:** 

Mechanism	Development	Agent	Code name	Phase	Target
SMI	*AVEO Pharmaceuticals*	Tivozanib	AV-951	III	VEGFR
	*Novartis*	Dovitinib	TKI258	I/II	FGFR, VEGFR, PDGFR
	*Bayer*	Regorafenib	BAY 73-4506	II	c-KIT, VEGFR, B-Raf.
	*Roche*	Linifanib	ABT-869	II	PDGF, VEGF
	*Recentin by AstraZeneca*	Cediranib	AZD-2171	II	VEGFR
	*Bristol-Myers Squibb*	Brivanib	BMS-540215	II	VEGFR, FGFR
Mab	*PDL BioPharma and Biogen Idec*	Volociximab	M200	II	α5β1 integrin
	*Anyara by Active Biotech*	Naptumomab estafenatox	ABR-217620	III	5T4
	*Medarex by Bristol-Myers Squibb*	Anti-PD-1	MDX-1106	I	PD-1
	*Dyax by ImClone Systems Inc.*	Ramucirumab	IMC-1121B	II	VEGFR
	*Amgen INC.*	Rilotumumab	AMG 102	II	HGF/SF
VDA	*Bionomics*		BNC105P	II	inhibits tubulin polimerisation
fusion protein against VEGF	*Zaltrapby Sanofi-Aventis& Regeneron Pharmaceuticals*	Aflibercept		II	VEGF, PGFL
peptibody	*Amgen INC*	Trebananib	AMG 386	II	angiopoietin 1/2

**Abbreviations**: adv RCC: advanced renal cell carcinoma breast cancer; CSF1R: Colony Stimulating Factor 1 Receptor; FGF: Fibroblast growth factor; FLT3: Fms-like tyrosine kinase receptor-3; GBM: glioblastoma multiforme; GIST: gastrointestinal stromal tumor; HCC: hepatocellular carcinoma; HGF/SF: Growth and motility factor hepatocyte growth factor/scatter factor; MAB: monoclonal antibody; mBC: metastatic breast cancer; mCRC: metastatic colorectal carcinoma; mTOR: Mammalian target of rapamycin; NSCLC: non small cell lung carcinoma; PD-1:Programmed death-1; PDGFR: Platelet-derived growth factor receptor; PNET: pancreatic neuroendocrin tumor; SEGA: subependymal giant cell astrocytoma; SMI: small molecular inhibitor; VDA: vascular disrupting agent; VEGFR: Vascular endothelial growth factor receptor.

**A:** 

First line																			
NCCN categ.		1					1			1		1			2A		2A		No treatment
Agent		Sunitinib					Tensirolimus			Pazopanib		Bevacizumab+INF			Sorafenib		HD IL2		
No. of patients		116	25	375	375	352	209		-	155	155	325	369	146	83		247	120	78
ORR	%	35.3	48	-	38.7	40.6	8.6		-	31	30	31	25.5	22	-	-	-	29	-
PFS	months	9	13.25	11	-	-	3.8	5.5	4.7	11	11.1	10.2	-	15.6	7.4	8.5	3.4	-	2.8
OS	months	-	35.85	26.4	28.7	-	10.9		-	-	-	23.9	18.3	-	-	-	15.3	-	-
Reference		[e]	[d]	[6]	[b]	[c]	[22]		^33^	[18]	[19]	[31]	[32, 33]	^34^	^35^		[37]	^36^	[19]

**B:** 

Second line																		
After TKI							After cytokin							No treatment				
NCCN categ.		1			-	2A	1		1			1						
Agent		Everolimus			Axitinib	Sorafenib	Sunitinib		Sorafenib			Pazopanib						
No. of patients		272	277	39	361	362	107	106	26	39	451	44	70	452	138	67	452	139
ORR	%	-	-	-	19	11	20	33	46	-	-	20	37	-	-	-	-	-
PFS	months	4	5.4	5.1	6.7	4.7	8.2	9.5	12	5.4	5.5	9.3	-	2.8	1.9	4.2	-	1.9
OS	months	8.8	-	-	-	-	20	26	35	7.8	19	-	-	16	8.8	-	14	-
Reference		[23]	^37,38^	^39^	[27]		[67]	[a] [4]	[d]	[f]	[10], [68]	^40^	[18]	[68]	[23]	[19]	[10]	^41^

Abbreviations: HD: high dose; NCCN: National Comprehensive Cancer Network; No.: number of; OS: overall survival; ORR: overall response rate; PFS: progression-free survival; TKI: tyrosine kinase inhibitors. *Letters: data of clinical trials:*

a: NCT00077974;

b: NCT00083889;

c: NCT00460798;

d: NCT00254540;

e: NCT00338884;

f: NCT00586105.

**Table 3.** Suggested predictive molecular biomarkers for renal cancer treatment.

Drug	Symbol	Full Name	pts #	Ref.
sunitinib	VEGF-A	Vascular endothelial growth factor A	38	^42^
	VEGF-C	Vascular endothelial growth factor C	61	[69]
	VEGF	Vascular endothelial growth factor	85	[70]
			42	[71]
			55	[72]
	sVEGFR-2	Vascular endothelial growth factor receptor 2		
	sVEGFR-3	Vascular endothelial growth factor receptor 3		
			61	[69]
	rs307826	rs307826: VEGFR-3 missense polymorphisms	89	[73]
	VEGF SNP 936 and VEGFR2 SNP 889	Combination of these two SNPs	63	[74]
	TNF-α	Tumor necrosis factor- α	31	[75]
	MMP-9	Matrix metallopeptidase 9		
	NGAL	Neutrophil gelatinase-associated lipocalin	85	[70]
	bFGF	Basic fibroblast growth factor	38	^42^
	IL-8	Interleukin-8	20	[76]
	HIF1A	Hypoxia-inducible factor 1	43	^43^
	HIF2A	Hypoxia-inducible factor 2		
	Peptides	Histones, Rho GTPase activating protein 29, CK1	15	^44^
sorafenib	VEGF	Vascular endothelial growth factor	712	[10, 77]
	CAIX	Carbonic anhydrase 9	94	[78]
pazopanib	sVEGFR-2	Vascular endothelial growth factor receptor 2	225	[79]
	IL-8 and HIF1A	Polymorphisms in IL-8 and HIF1A	397	[80]
	IL-8	Interleukin-8	129	^45^
	IL-6	Interleukin-6	225	^46^
	OPN	Osteopontin		
	HGF	Hepatocyte growth factor		
			129	^47^
temsirolimus	LDH	Lactate dehydrogenase	404	[81]
	pS6	Phospho-S6	20	[82]
VEGF targeted therapy *	VHL	Loss of function mutations of VHL	123	[83]
IL-2	CAIX	Carbonic anhydrase 9	66	[84]
	VEGF and FN1	Vascular endothelial growth factor and Fibronectin	60	[85]
	Bcl-2 and Fas	B-cell CLL/lymphoma 2 and Fas	40	[86]
IFN-α and low-dose IL-2	Bcl-2	B-cell CLL/lymphoma 2	40	[87]
celecoxib and IFN- α	COX-2	Cyclooxygenase-2	25	[88]

* sunitinib, sorafenib, axitinib and bevacizumab

**Abbreviations:** VEGF: Vascular endothelial growth factor, sVEGFR-2: Vascular endothelial growth factor receptor 2 in serum, TNF-α: Tumor necrosis factor-α, MMP-9: Matrix metallopeptidase 9, NGAL: Neutrophil gelatinase-associated lipocalin, bFGF: Basic fibroblast growth factor, IL-8: Interleukin-8, HIF: Hypoxia-inducible factor, CK1: casein kinase 1, CAIX: Carbonic anhydrase 9, IL-6: Interleukin-6, OPN: Osteopontin, HGF: Hepatocyte growth factor, LDH: Lactate dehydrogenase, pS6: Phospho-S6, VHL: von Hippel-Lindau tumor suppressor, FN1: Fibronectin, Bcl-2: B-cell CLL/lymphoma 2.

## Footnotes

1 Pickering AC, J. M. L., S. A. Sohaib, P. Khatri, P. D. Nathan, A. Vinayan, T. Ferguson, S. St. Rose and M. E. Gore. Sunitinib treatment for longer than 2 years for renal cell carcinoma. *J. Clin. Oncol*. **2010**, 28, abstr 15099.

2 Casper, D. G., V. Gruenwald, A. Flörcken, D. Mueller, K. Toussaint, B. MetznerJ. C. Efficacy and safety of sunitinib in patients with metastatic renal cell carcinoma on hemodialysis. *J. Clin. Oncol*. **2011**, 29, abstr 4646.

3 Gore, R. J. J., A. Ravaud, M. Kuczyk, T. Demkow, A. Bearz, N. Laferriere, U. P. Strauss, C. Porta. Efficacy and safety of intrapatient dose escalation of sorafenib as first-line treatment for metastatic renal cell carcinoma (mRCC). *J. Clin. Oncol*. **2011**, 29, abstr 4609.

4 Mancuso, E. D. D. P., A. Catalano, F. Calabrò, C. Messina, A. Zivi, L. Cerbone, L. Vigna, R. Caristo, C. N. Sternberg. Phase II dose escalation study of sorafenib in patients with metastatic renal cell carcinoma (mRCC) who have had prior treatment with VEGFR-TKI antiangiogenic treatment. *J. Clin. Oncol*. **2009**, 27, abstr 16027.

5 Waterhouse, W. C. P., C. D. Webb, F. A. Greco, H. A. Burris, J. D. Hainsworth Sorafenib and Everolimus (RAD001) in the treatment of patients with advance renal clear cell renal carcinoma (RCC): A Sarah Cannon Research Institute phase I/II trial. *J. Clin. Oncol*. **2011**, 29, abstr 4629.

6 Rini, B. E., P. Tomczak, A. Kaprin, T. E. Hutson, C. Szczylik, J. C. Tarazi, B. Rosbrook, S. Kim, R. J. Motzer, Axitinib versus sorafenib as second-line therapy for metastatic renal cell carcinoma (mRCC): Results of phase III AXIS trial. *J. Clin. Oncol* **2011**, 29, abstr 4503.

7 Casciano EM, S. S., J. Baladi, A. C. Kay, D. Kim, C. A. Garay, N. Hollaender and J. Wang. An indirect comparison of everolimus and sorafenib therapy in sunitinibrefractory mRCC patients. *J. Clin. Oncol*. **2010**, 28, abstr 4611.

8 Rini CS, N. M. T., P. Koralewski, P. Tomczak, A. Deptala, K. Kracht, Y. Sun, M. Puhlmann and B. Escudier MG 386 in combination with sorafenib in patients (pts) with metastatic renal cell cancer (mRCC): A randomized, double-blind, placebo-controlled, phase II study. *J. Clin. Oncol*. **2011**, 29, abstr 309.

9 McCann L., O. Amit, L. Pandite, R. G. Amado. An indirect comparison analysis of pazopanib versus other agents in metastatic renal cell carcinoma (mRCC). *J. Clin. Oncol*. **2010**, 28, abstr 15128.

10 J. P. Dutcher, C. S., N. Tannir, P. Benedetto, P. Ruff, A. Hsu, A. Berkenblit, A. Thiele, A. Strahs, J. Feingold. Correlation of survival with tumor histology, age, and prognostic risk group for previously untreated patients with advanced renal cell carcinoma (adv RCC) receiving temsirolimus (TEMSR) or interferon-alpha (IFN). *J. Clin. Oncol*. **2007**, 25, abstr 5033.

11 Pelz H GK, L. B., Sr., J. Roigas, T. Steiner, P. Löschmann and M. Kosch. Evaluation of safety, tolerability, and activity: A registry for temsirolimus-treated patients with advanced or metastatic renal cell carcinoma (aRCC) in the usual health care setting. . *J. Clin. Oncol*. **2010**, 28, abstr 15127.

12 Kay, R. M., R. Figlin, B. Escudier, S. Oudard, C. Porta, T. Hutson, S. Bracarda, N. Hollaender, G. Urbanowitz, A. Ravaud. Updated data from a phase III randomized trial of everolimus (RAD001) versus PBO in metastatic renal cell carcinoma (mRCC). *Genitourinary Cancers Symposium* **2009**, abstr 278.

13 Figlin EC, R. J. M., T. E. Hutson, S. Oudard, C. Porta, V. Grunwald, A. Ravaud and E. Kpamegan. Everolimus in metastatic renal cell carcinoma (mRCC): Subgroup analysis of patients (pts) with one versus two prior vascular endothelial growth factor receptor tyrosine kinase inhibitor (VEGFR-TKI) therapies enrolled in the phase III RECORD-1 study. *J. Clin. Oncol*. **2011**, 29, abstr 304.

14 Gruenwald, M. F., C. Seidel, M. Heuser, A. Ganser. Antitumor activity of sequential treatment with tyrosine kinase inhibitors (TKI) after failure of RAD001 in metastatic renal cell carcinoma (mRCC). *J. Clin. Oncol*. **2010**, 28, abstr e15132.

15 Whorf, J. D. H., D. R. Spigel, D. A. Yardley, H. A. Burris, III, D. M. Waterhouse, E. R. Vazquez, F. A. Greco. Phase II study of bevacizumab and everolimus (RAD001) in the treatment of advanced renal cell carcinoma (RCC). *J. Clin. Oncol*. **2008**, 26, abstr 5010.

16 Rini, B. E., P. Tomczak, A. Kaprin, T. E. Hutson, C. Szczylik, J. C. Tarazi, B. Rosbrook, S. Kim, R. J. Motzer, Axitinib versus sorafenib as second-line therapy for metastatic renal cell carcinoma (mRCC): Results of phase III AXIS trial. *J. Clin. Oncol* **2011**, 29, abstr 4503.

17 Tomita Y., H. Uemura, H. Fujimoto, H. Kanayama, N. Shinohara,H. Nakazawa, S. Ozono, S. Naito, H. Akaza. Key predictive factors of axitinib (AG-013736)-induced proteinuria and efficacy: a Japanese phase II study in patients with cytokinerefractory metastatic renal cell cancer (mRCC). In *35th Annual Congress of the European Society for Medical Oncology (ESMO)*, Milan, **2010**; abstr. 902P.

18 Melichar, S. B., V. Matveev, I. Rusakov, A. Kaprin, A. Zyryanov, R. Janciauskiene, E. Fernebro, G. H. Mickisch, M. E. Gore, A. Schulze, S. Jethwa, V. Sneller, P. Mulders, J. Bellmunt. BEVLiN: Prospective study of the safety and efficacy of firstline bevacizumab (BEV) plus low-dose interferon-α2a (IFN) in patients (pts) with metastatic renal cell carcinoma (mRCC). *J. Clin. Oncol*. **2011**, 29, abstr 4546.

19 Likun, Y. B. A meta-analysis of bevacizumab plus interferon alfa for previously untreated patients with metastatic clear-cell renal carcinoma (mRCC). *J. Clin. Oncol*. **2011**, 29, abstr 366.

20 Halabi, B. I. R., W. M. Stadler , E. J. Small. Use of progression-free survival (PFS) to predict overall survival (OS) in patients with metastatic renal cell carcinoma (mRCC). *J. Clin. Oncol*. **2010**, 28, abstr 4525.

21 Azzi, F. S., Y. Je and T. K. Choueiri. Bevacizumab (BEV) and the risk of arterial thromboembolic events (ATE) in patients with renal cell carcinoma and other cancers: A large comprehensive meta-analysis of more than 13,000 patients. *J. Clin. Oncol*. **2010**, 28, abstr 4609.

22 Bukowski, F. K., R. A. Figlin, K. Flaherty, S. Srinivas, U. Vaishampayan, H. Drabkin, J. Dutcher, F. Scappaticci, D. McDermott. Bevacizumab with or without erlotinib in metastatic renal cell carcinoma (RCC). *J. Clin. Oncol*. **2006**, 24, abstr 4523.

23 McDermott MSG, S. S., K. A. Margolin, J. Clark, J. A. Sosman, J. P. Dutcher, T. Logan, R. A. Figlin, M. B. Atkins and Cytokine Working Group. The high-dose aldesleukin (HD IL-2) "SELECT" trial in patients with metastatic renal cell carcinoma (mRCC). *J. Clin. Oncol*. **2010**, 28, abstr 4514.

24 Bhargava BE, D. A. N., O. N. Lipatov, A. A. Lyulko, A. A. Anischenko, R. T. Chacko, P. Lee, M. Al-Adhami and J. Ryan. Updated activity and safety results of a phase II randomized discontinuation trial (RDT) of AV-951, a potent and selective VEGFR1, 2, and 3 kinase inhibitor, in patients with renal cell carcinoma (RCC). *J. Clin. Oncol*. **2009**, 27, abstr 5032.

25 Nosov, P. B., W. B. Esteves, A. L. Strahs, O. N. Lipatov, O. O. Lyulko, A. O. Anischenko, R. T. Chacko, D. Doval, W. J. Slichenmyer. Final analysis of the phase II randomized discontinuation trial (RDT) of tivozanib (AV-951) versus placebo in patients with renal cell carcinoma (RCC). *J. Clin. Oncol*. **2011**, 29, abstr 4550.

26 Motzer RJ, P. B., B. Esteves, M. Al-Adhami, W. Slichenmyer, D. Nosov, T. Eisen, C. N. Sternberg, T. E. Hutson. A phase III, randomized, controlled study to compare tivozanib with sorafenib in patients (pts) with advanced renal cell carcinoma (RCC). *J. Clin. Oncol*. **2011**, 29, abstr. 310.

27 Angevin, V. G., A. Ravaud, D. E. Castellano, C. C. Lin, J. E. Gschwend, A. L. Harzstark, J. Chang, Y. Wang, M. M. Shi, B. J. Escudier. A phase II study of dovitinib (TKI258), an FGFR- and VEGFR-inhibitor, in patients with advanced or metastatic renal cell cancer (mRCC). *J. Clin. Oncol*. **2011**, 29, abstr 4551.

28 Eisen, H. J., P. Nathan, P. Harper, M. Wojtukiewicz, S. Nicholson, A. Bahl, P. Tomczak, A. Wagner, D. Phase II study of BAY 73-4506, a multikinase inhibitor, in previously untreated patients with metastatic or unresectable renal cell cancer. *J. Clin. Oncol*. **2009**, 27, abstr 5033.

29 Tannir, Y. W., C. K. Kollmannsberger, M. S. Ernstoff, D. J. Perry, L. J. Appleman, E. M. Posadas, J. Qian, J. L. Ricker, D. Michaelson. Phase II trial of linifanib in patients with advanced renal cell cancer (RCC) after sunitinib failure. *J. Clin. Oncol*. **2010**, 28, abstr 4527.

30 Sridhar, M. J. M., S. J. Hotte, S. D. Mukherjee, C. Kollmannsberger, M. A. Haider, E. X. Chen, L. Wang, R. Srinivasan, S. P. Ivy, M. J. Moore. Activity of cediranib (AZD2171) in patients (pts) with previously untreated metastatic renal cell cancer (RCC). A phase II trial of the PMH Consortium. *J. Clin. Oncol*. **2008**, 26, abstr 5047.

31 Yazji S., R. B., V. Kondagunta, R. Figlin. Final results from phase II study of volociximab, an α5β1 anti-integrin antibody, in refractory or relapsed metastatic clear cell renal cell carcinoma (mCCRCC). *J. Clin. Oncol* **2007**, 25, abstr 5094.

32 Rini, C. S., N. M. Tannir, P. Koralewski, P. Tomczak, A. Deptala, K. Kracht, Y. Sun, M. Puhlmann and B. Escudier. AMG 386 in combination with sorafenib in patients (pts) with metastatic renal cell cancer (mRCC): A randomized, double-blind, placebocontrolled, phase II study. *J. Clin. Oncol*. **2011**, 29. Abstr. 309

33 Pelz H GK, L. B., Sr., J. Roigas, T. Steiner, P. Löschmann and M. Kosch. Evaluation of safety, tolerability, and activity: A registry for temsirolimus-treated patients with advanced or metastatic renal cell carcinoma (aRCC) in the usual health care setting. *J. Clin. Oncol*. **2010**, 28, abstr 15127.

34 Melichar, S. B., V. Matveev, I. Rusakov, A. Kaprin, A. Zyryanov, R. Janciauskiene, E. Fernebro, G. H. Mickisch, M. E. Gore, A. Schulze, S. Jethwa, V. Sneller, P. Mulders, J. Bellmunt. BEVLiN: Prospective study of the safety and efficacy of first-line bevacizumab (BEV) plus low-dose interferon-α2a (IFN) in patients (pts) with metastatic renal cell carcinoma (mRCC). *J. Clin. Oncol*. **2011**, 29, abstr 4546.

35 Gore, R. J. J., A. Ravaud, M. Kuczyk, T. Demkow, A. Bearz, N. Laferriere, U. P. Strauss, C. Porta. Efficacy and safety of intrapatient dose escalation of sorafenib as first-line treatment for metastatic renal cell carcinoma (mRCC). *J. Clin. Oncol*. **2011**, 29, abstr 4609.

36 McDermott MSG, S. S., K. A. Margolin, J. Clark, J. A. Sosman, J. P. Dutcher, T. Logan, R. A. Figlin, M. B. Atkins and Cytokine Working Group. The high-dose aldesleukin (HD IL-2) "SELECT" trial in patients with metastatic renal cell carcinoma (mRCC). *J. Clin. Oncol*. **2010**, 28, abstr 4514.

37 Kay, R. M., R. Figlin, B. Escudier, S. Oudard, C. Porta, T. Hutson, S. Bracarda, N. Hollaender, G. Urbanowitz, A. Ravaud. Updated data from a phase III randomized trial of everolimus (RAD001) versus PBO in metastatic renal cell carcinoma (mRCC). *Genitourinary Cancers Symposium* **2009**, abstr 278.

38 Figlin EC, R. J. M., T. E. Hutson, S. Oudard, C. Porta, V. Grunwald, A. Ravaud and E. Kpamegan. Everolimus in metastatic renal cell carcinoma (mRCC): Subgroup analysis of patients (pts) with one versus two prior vascular endothelial growth factor receptor tyrosine kinase inhibitor (VEGFR-TKI) therapies enrolled in the phase III RECORD-1 study. *J. Clin. Oncol*. **2011**, 29, abstr 304.

39 Gruenwald, M. F., C. Seidel, M. Heuser, A. Ganser. Antitumor activity of sequential treatment with tyrosine kinase inhibitors (TKI) after failure of RAD001 in metastatic renal cell carcinoma (mRCC). *J. Clin. Oncol*. **2010**, 28, abstr e15132.

40 Rini CS, N. M. T., P. Koralewski, P. Tomczak, A. Deptala, K. Kracht, Y. Sun, M. Puhlmann and B. Escudier. MG 386 in combination with sorafenib in patients (pts) with metastatic renal cell cancer (mRCC): A randomized, double-blind, placebo-controlled, phase II study. *J. Clin. Oncol*. **2011**, 29, abstr 309.

41 Figlin EC, R. J. M., T. E. Hutson, S. Oudard, C. Porta, V. Grunwald, A. Ravaud and E. Kpamegan. Everolimus in metastatic renal cell carcinoma (mRCC): Subgroup analysis of patients (pts) with one versus two prior vascular endothelial growth factor receptor tyrosine kinase inhibitor (VEGFR-TKI) therapies enrolled in the phase III RECORD-1 study. *J. Clin. Oncol*. **2011**, 29, abstr 304.

42 Tsimafeyeu I, Ta H, Stepanova E, Wynn N. Fibroblast growth factor pathway in renal cell carcinoma. *J. Clin. Oncol*. **2010**;28(15S) abstr.372s

43 Patel, P. H.; Chadalavada; Ishill, N.; Patil, S.; Reuter, C. W.; Motzer, R. Chaganti. In Hypoxia-inducible factor (HIF) 131. Escudier, B.; Pluzanska, A.; Koralewski, P.; Ravaud, A.; Bracarda, S.; Szczylik, C.; Chevreau, C.; Filipek, M.; Melichar, B.; Bajetta, E.; Gorbunova, V.; Bay, J. O.; Bodrogi, I.; Jagiello-Gruszfeld, A.; Moore, N.; investigators, A. T. Bevacizumab plus interferon alfa-2a for treatment of metastatic renal cell carcinoma: a randomised, double-blind phase III trial. Lancet. 2007, 370 (9605), 2103-2111. and 231. Ibid. levels in cell lines and human tumor predicts response to sunitinib in renal cell carcinoma (RCC). *ASCO Annual Meeting Proceedings*, **2008**; p 5008.

44 Gámez-Pozo A., A. A. L., Gallegos I., Sánchez-Navarro I., Borrega P., Bayona C., Ramos M. Biomarkers predicting major response to sunitinib in metastatic renal cell carcinoma. *2010 Genitourinary Cancers Symposium* 381

45 Heymach J, T. H., Fritsche HA. Lower baseline levels of plasma hepatocyte growth factor (HGF), IL-6 and IL-8 are correlated with tumor shrinkage in renal cell carcinoma patients treated with pazopanib. In *AACR-NCI-EORTC International Conference on Molecular Targets and Cancer Therapeutics*, Boston, MA, 2009.

46 Liu, Y. Circulating Biomarkers in Pazopanib Phase III Trial. In *ASCO 2011 Reports*.

47 Heymach J, T. H., Fritsche HA. Lower baseline levels of plasma hepatocyte growth factor (HGF), IL-6 and IL-8 are correlated with tumor shrinkage in renal cell carcinoma patients treated with pazopanib. In *AACR-NCI-EORTC International Conference on Molecular Targets and Cancer Therapeutics*, Boston, MA, 2009.

## ABBREVIATIONS

ASCO: = American Society of Clinical Oncology
CI: = Confidence interval
EAU: = European Association of Urology
ErbB: = Erythroblastic Leukemia Viral Oncogene Homolog
FDA: = Food and Drug Administration
IFN: = Interferon α
mRCC: = Metastatic renal cell carcinoma
mTOR: = Mammalian target of rapamycin
NCCN: = National Comprehensive Cancer Network
OS: = Overall survival
ORR: = Overall response rate
PD-1: = Programmed death-1
PFS: = Progression-free survival
QUALY: = Quality Adjusted Life Years
RAS: = Rat sarcoma
RCC: = Renal cell carcinoma
RECIST: = Response Evaluation Criteria in Solid Tumors
TKI: = Tyrosine kinase inhibitors
VEGF: = Vascular endothelial growth factor
